# Motor skill experience modulates attentional processing regardless of open- or closed-skill types: an ERP study

**DOI:** 10.3389/fpsyg.2024.1460684

**Published:** 2024-12-20

**Authors:** Mohamed Aly, Turki Alzahrani, Mohammed Fakehy, Mohamed Abass, Sheref Mohamed

**Affiliations:** ^1^Department of Educational Sciences and Sports Psychology, Faculty of Physical Education, Assiut University, Assiut, Egypt; ^2^Department of Sport Sciences, College of Education, Taif University, Taif, Saudi Arabia; ^3^Department of Biomechanics and Motor Behavior, College of Sport Sciences and Physical Activity, King Saud University, Riyadh, Saudi Arabia

**Keywords:** aerobic fitness, sports activity, open-skill exercise, closed-skill exercise, exercise mode, event-related potential

## Abstract

**Background:**

Attentional engagement, the ability to maintain focus on relevant tasks, plays a crucial role in optimizing human performance. Studies have shown that athletes exhibit superior attentional engagement compared to non-athletes; however, it remains unclear if these benefits persist in non-sport-related tasks or differ across types of sports expertise, such as open-skill versus closed-skill sports.

**Methods:**

Ninety-three young adults, divided into open-skill athletes (*n* = 31), closed-skill athletes (*n* = 31), and a control group (*n* = 31), completed an auditory oddball task while the P3 component of event-related potentials was measured to assess attentional processing. Aerobic fitness was assessed using the YMCA fitness test, and linear regression models, adjusted for confounders, examined the relationship between aerobic fitness and attentional processing.

**Results:**

Findings revealed that both open- and closed-skill athletes exhibited significantly larger P3 amplitudes than non-athletes, indicating enhanced attentional engagement. However, no significant differences in response time or response accuracy were observed between the groups. Hierarchical regression analysis further demonstrated a positive correlation between aerobic fitness and P3 amplitude, underscoring the role of aerobic fitness in cognitive processing.

**Conclusion:**

These findings suggest that participation in either open- or closed-skill sports can enhance attentional engagement beyond the sports context in healthy young adults, with aerobic fitness playing a key role in supporting cognitive performance. Additionally, this study extends previous findings from Western and Asian literature by providing evidence from an Egyptian sample, thereby supporting the generalizability of the cognitive benefits of aerobic fitness and sports participation across different cultural contexts.

## Introduction

The interplay between motor experience and cognitive functions has garnered significant interest within the realms of sports science and neuroscience (Moreau, 2015). Research has shown that cognitive functions, such as attention, executive control, and working memory, can benefit from sports practice and long-term sport-related training (Abdelkarim et al., 2023; Aly et al., 2023; Gutiérrez-Capote et al., 2024). For instance, Voss et al. (2010) provide a comprehensive review indicating a positive relationship between motor training and cognitive performance. However, a critical question remains: does expertise in specific sports disciplines enhance performance on general cognitive tasks unrelated to the sports context? Investigating this question could provide deeper insights into how sports practice influences cognitive processes beyond the sports setting. Such understanding may also offer valuable guidance for athletic programs and physical education curricula. For instance, if certain types of sports are linked to more significant cognitive benefits, educators and public health officials could advocate for these activities among adolescents, individuals with cognitive deficits, and the elderly. While studies demonstrate that elite athletes show superior proficiency in cognitive tasks involving problem-solving, motor planning, and decision-making compared to non-athletes (Vestberg et al., 2012), it remains unclear how these benefits vary across different types of sports. This observed cognitive advantage aligns with the cognitive skill transfer theory, which posits a ‘broad transfer’ mechanism whereby the intensive practice of specific skills, such as those developed through sports, enhances cognitive components utilized beyond the sports context (Taatgen, 2013). Furley and Memmert (2011) support this theory, noting that sports training can improve individual cognitive functions applicable in various non-sport settings. In terms of attentional functioning, studies have shown that athletes perform better in attention-demanding tasks than non-athletes, with differences in brain activation and deactivation patterns (Bianco et al., 2017; Faubert, 2013; Qiu et al., 2019).

While it is established that athletes exhibit enhanced attentional processing (Vestberg et al., 2012), limited research has explored whether the type of sport, particularly open- versus closed-skill sports, influences the degree of attentional enhancement. The cognitive and motor demands differ significantly between sports, which may differently influence attentional processes (Nakata et al., 2010; Overney et al., 2008; Yarrow et al., 2009). Sports can generally be divided into two categories: open skill and closed skill. Open-skill sports, such as basketball, tennis, and fencing, require athletes to respond to a constantly changing, unpredictable environment (Di Russo et al., 2010). Conversely, closed-skill sports like running and swimming take place in more predictable, consistent settings (Di Russo et al., 2010; Voss et al., 2010). Athletes in open-skill sports often develop greater flexibility in visual attention, decision-making, and action execution compared to their counterparts in closed-skill sports due to the complex cognitive demands of adapting to an ever-changing environment (Taddei et al., 2012).

Previous research suggests that open-skill sports may lead to greater enhancements in cognitive functions due to the complex cognitive demands of adapting to dynamic environments (Ingold et al., 2020; Takahashi and Grove, 2023; Zhou et al., 2020). This distinction is underscored by meta-analyses showing that athletes from open-skill sports perform better on cognitive tasks than those from closed-skill sports, highlighting the importance of comparing sport types (Mann et al., 2007; Voss et al., 2010). However, most studies have assessed these cognitive benefits using tasks that require high levels of cognitive control, such as inhibitory control (e.g., Stop Signal Task; Wang et al., 2013), working memory (e.g., Digit Span Backward Test; Russo et al., 2021), visuospatial working memory (e.g., Corsi’s Block Tapping Test; Gökçe et al., 2021; Guo et al., 2016), interference control (e.g., Stroop Task; Zhou et al., 2020), cognitive flexibility (e.g., Task Switching Task; Yu et al., 2017), visuospatial attention and memory processing (e.g., non-delayed and delayed match-to-sample tasks; Chueh et al., 2017), and visuospatial attention (e.g., line-length judgment task; Giglia et al., 2011). Furthermore, the majority of these studies have focused on behavioral outcomes, with relatively few investigating the underlying neurophysiological mechanisms. As a result, there is limited understanding of the neuroelectric processes that may differentiate cognitive benefits in open-skill versus closed-skill sports.

While behavioral studies offer insights into the cognitive benefits observed in athletes, they do not adequately probe the neural mechanisms that support these benefits. Deriving from electroencephalogram (EEG), P3 (also known as P300) is a positive ongoing deflection of brain potential peaking between 300 and 800 ms after the stimulus onset and indicates the updating of mental representation as a result of top-down controlled attention reacting to a bottom-up stimulus (Polich, 2007). P3 has widespread and synchronous neural generators centered at the cingulate cortex linking frontal-mediated attention and updating to memory storage operated in the temporal/parietal brain areas (Kim, 2014; Soltani and Knight, 2000). The neural substrates of P3 correspond with the attentional processing. P3 can be elicited during tasks requiring attentional processes (e.g., auditory oddball task), with increasing amplitude reflecting greater attentional resources allocated to processing the target stimulus and decreasing latency reflecting a faster speed of stimulus categorization (Luck and Kappenman, 2011; Polich, 2007). More efficient allocation of attentional resources to the stimulus, as reflected by larger P300 amplitudes, was reported for all regular exercisers compared with those who did not exercise regularly (Dai et al., 2013; Huang et al., 2014). Previous research utilizing ERPs has also indicated that prolonged training in sports demanding rapid responses to dynamic environments may foster several neurocognitive enhancements. These enhancements include additional cognitive benefits in areas such as inhibitory control and error processing, as evidenced by decreased N2 and increased P3 amplitudes (Li et al., 2019); heightened task-oriented attention, indicated by increased P3 amplitude (Aly et al., 2019; Aly and Kojima, 2020b; John and Lardon, 1997); and more efficient cognitive processing, reflected by shorter P3 latency (Gostilovich et al., 2023).

ERP studies have consistently demonstrated that open-skill sports, as compared to closed-skill sports, are associated with enhanced cognitive performance and neural efficiency, particularly in older adults. These benefits are often observed in areas such as inhibitory control, cognitive flexibility, and visuospatial attention, as evidenced by increased P3 amplitudes in ERP measurements (Dai et al., 2013; Huang et al., 2014; Li et al., 2019; Tsai et al., 2016; Tsai and Wang, 2015). For example, open-skill exercisers display faster response times, improved inhibitory control, and heightened error processing in tasks like Stroop and task-switching, reflecting better cognitive flexibility and attentional control (Li et al., 2019; Tsai et al., 2016; Tsai and Wang, 2015) wang. Additionally, open-skill sports appear to promote more efficient resource allocation for tasks requiring high cognitive control, as seen in larger P3 amplitudes (Dai et al., 2013; Huang et al., 2014). These findings suggest that the complex and adaptive demands of open-skill sports may confer greater cognitive benefits than closed-skill sports, particularly in enhancing attentional and executive functions.

The purpose of this study was to examine the relationship between motor skill type and attentional processing efficiency in young adults using behavioral and neuroelectric measures. Given that previous research suggests that sports incorporating higher attentional demands can enhance attentional functioning (Zhu et al., 2020), we hypothesized that individuals who regularly engage in physical exercise would exhibit shorter response time, shorter P3 latency, and increased P3 amplitude compared to those who exercise irregularly (i.e., control). We also expected that participants involved in open-skill sports would demonstrate shorter response times, shorter P3 latency, and larger P3 amplitude than those engaged in closed-skill sports. The findings of this study are expected to advance our understanding of the generalizable cognitive benefits of motor skill experience and have practical implications for designing effective training and rehabilitation programs.

## Materials and methods

### Participants

Ninety-three participants were recruited from Assiut University and classified into three groups: open-skilled, closed-skilled, and control participants (Table 1). All participants were right-handed, with normal or corrected-to-normal visual acuity, and had no history of neurological disorders, cardiovascular diseases, or clinical conditions requiring medication. None of the participants showed cognitive impairments (a score of below 27 on the Mini-Mental State Examination, MMSE) (Al-Rajeh et al., 1999; Folstein et al., 1975) or depressive symptoms (a score of above 13 on the Beck Depression Inventory-II [BDI-II]) (Alansari, 2006; Beck et al., 1996). The participants with expertise in open and closed skills were members of university sports teams and Assiut city sports clubs, with at least 5 years of professional training. They had won prizes in open competitions in their respective sports but had not received regular training in other sports. Among these, the open-skilled participants (*n* = 31; 10 females) were from football and handball teams, and the closed-skilled participants (*n* = 31; 12 females) were from swimming and track and field teams. The control group (*n* = 31; 12 females) consisted of university students who had never participated in sports either professionally or at an amateur level. All three groups were age-matched. Each participant provided written informed consent. Ethical approval was received from the Institutional Review Board at the Faculty of Physical Education, Assiut University (No. AUN-PHY-JUN-ETHIC2), with all methods undertaken thereafter performed in accordance with the relevant guidelines and regulations.

**Table 1:** Descriptive data for participants’ demographic and physical characteristics among three groups (mean ± 1SD).

	Open-skill (*n* = 31)	Closed-skill (*n* = 31)	Control (*n* = 31)
Female (*x*^2^)	10	12	12
Age (yr)	19.45 ± 1.36	19.51 ± 1.31	19.61 ± 1.50
Education (yr)	12.55 ± 1.29	12.62 ± 1.07	12.52 ± 1.39
Height (M)	1.75 ± 0.06	1.76 ± 0.07	1.73 ± 0.06
Weight (kg)	70.02 ± 6.87	69.37 ± 7.06	73.27 ± 7.91
MMSE (score)	29.03 ± 1.01	29.26 ± 0.81	29.10 ± 0.86
BDI (score)	3.32 ± 1.62	3.35 ± 1.87	3.12 ± 1.69
BMI (kg/m^2^)	22.85 ± 1.37	22.48 ± 1.54	24.36 ± 2.34
Exercise experience			
Years (regular)	7.03 ± 1.23	7.10 ± 1.15	0.72 ± 0.27
Duration/session (min)	96.77 ± 22.86	92.90 ± 24.93	7.74 ± 17.26
Frequency/week	3.03 ± 0.84	2.97 ± 0.95	0.19 ± 0.40
IPAQ (METs)	3,792.45 ± 506.84	3,813.65 ± 384.84	2,257.23 ± 527.77
YMCA (VO2peak)	53.18 ± 9.579	54.23 ± 8.399	29.20 ± 5.53

MMSE, mini-mental state exam; BDI, Beck Depression Inventory (scores ≥ 14 are suggestive of clinically significant depressive symptoms); BMI, body mass index; IPAQ, International physical activity questionnaire; METs, metabolic equivalents. *x*^2^, variables compared with chi-square.

### Electroencephalogram recordings

EEG was measured using surpass EMS biomedical, quantitative EMG/EP work station. The neuroelectric assessment was done by applying an auditory oddball task. The auditory stimuli had 10 ms r/f time and were either a 1,000 Hz (frequent) or 2,000 Hz (rare) tone (100 ms duration/80 dB SPL, 1.2–1.7 interstimulus interval), delivered binaurally through headphones and completely covering the ears (Polich, 1998). The order of stimulus presentation was randomized, with a 1-s inter-stimulus interval. Event related evoked potentials were recorded with Ag/ Ag Cl (Nihon Kohden) electrodes from standard locations using a 10–20 International system. The electrodes were placed at Fz, Cz, and Pz (active electrodes at frontal, vertex, and parietal areas), FPz (ground electrode on the forehead), and A1, A2 (reference electrode on the ear lobules). The vertical and horizontal eye movement artifacts (VEOG and HEOG, respectively) were assessed through the collection of bipolar electro-oculographic activity (EOG). The impedances were maintained below 10 kΩ. The measured variables include P3 amplitude measured in microvolt and latency measured in milliseconds. Each participant was instructed to respond to the occurrence of a rare stimulus by pressing a button with the thumb of their preferred hand, and response time and response error rates were recorded. Brain responses to rare stimuli were recorded and averaged. P3 was quantified as the most positive peak amplitude between 250 and 500 msec. A practice session was conducted to ensure comprehension of the task followed by the test session.

### Aerobic fitness assessment

Aerobic fitness was assessed using the YMCA cycle ergometry protocol, which was recommended for submaximal exercise testing by the American College of Sports Medicine (2013). This protocol estimates maximal oxygen consumption (VO2peak) with an electronically braked cycle ergometer (Monark Ergomedic 828E). Participants engaged in two to four 3-min cycling stages, maintaining a pedal speed of 50 rpm throughout the assessment. Initially, a workload of 0.5 kp was applied, and heart rates were monitored in the last 15–30 s of this stage. These preliminary heart rate readings were used to adjust the workloads for subsequent stages (for example, heart rates above 80 bpm led to workloads of 2.5 kp and 3.0 kp for the second and third stages, respectively; heart rates above 100 bpm resulted in workloads of 1.5 kp and 2.0 kp). The protocol concluded once participants maintained heart rates between 110 and 150 bpm during the latter stages. Additionally, both objective and subjective measures of exercise intensity were employed, utilizing a Polar heart rate monitor (Sport Tester PE 3000, Polar Electro Oy, Kempele, Finland) and the Borg 6- to 20-point Rating of Perceived Exertion (RPE) scale, respectively (Borg, 1998). VO2peak was calculated using a graphical method prediction equation that takes into account average heart rate, workload, age, and gender (Heyward and Gibson, 2014).

### Procedure

**Figure 1:**
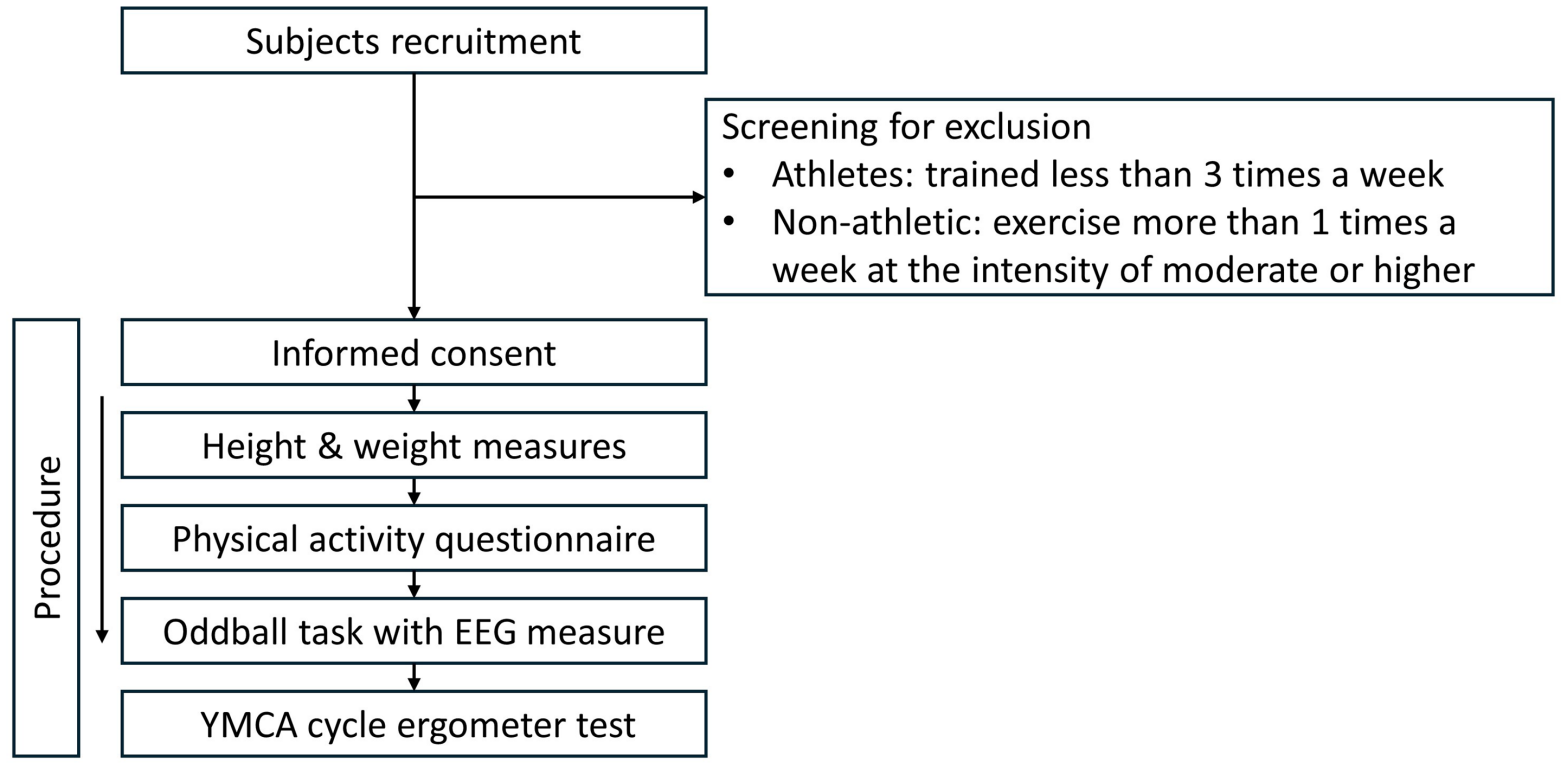
Study procedure and participant screening process. The flowchart illustrates the sequential steps in the study protocol, including subject recruitment, screening criteria for exclusion, informed consent, initial measurements (height, weight), completion of the physical activity questionnaire, the auditory oddball task with EEG measurement, and the YMCA cycle ergometer test.

In this cross-sectional study, participants were required to visit the laboratory for a single-day session, with instructions to refrain from exercising the day before their scheduled testing. Upon arrival, they provided informed consent and completed a demographics questionnaire, MMSE (Al-Rajeh et al., 1999; Folstein et al., 1975), Beck Depression Inventory-II (BDI-II) (Alansari, 2006; Beck et al., 1996), as well as the Physical Activity Readiness Questionnaire (Thomas et al., 1992). This was to identify any existing health conditions that could potentially be aggravated by the aerobic fitness assessment. Subsequently, participants were equipped with a heart rate monitor, and their height and weight were measured using a stadiometer and a digital scale (Omron Healthcare, Inc., Lake Forest, IL, USA), respectively. Following these measurements, participants were made to sit comfortably in a chair, whose back was turned toward the EEG recording machine in a semi-dark room with quiet surroundings. The participants were asked to avoid unnecessary movement and to remove all the metallic ornaments that they were wearing. After that, they performed the auditory oddball task. The participants’ aerobic fitness was assessed using a test of YMCA submaximal fitness test following the completion of the auditory oddball task to ensure that attentional functioning was not altered as a result of the fitness test (Pontifex et al., 2019). The experimental procedure is illustrated in Figure 1.

### Statistical analysis

All subjects’ demographic characteristics (i.e., age, years of education, amount of physical activity) and the behavioral (i.e., response time and response accuracy) and neuroelectric (i.e., P3 amplitude and latency) were described using means and SDs for each group (i.e., open-skill exercisers, closed-skill exercisers, control).

To confirm Pz as the region of interest to examine P3 (amplitude and latency) measures during the attentional processes in response to target stimuli, a two-way 2 (Stimulus: Target, Standard) × 3 (Electrode: Fz, Cz, Pz) repeated measure ANOVA was conducted to verify the target-specific and parietal-centered P3. Group differences in behavioral and neuroelectric measures were submitted to a one-way ANOVA method with a post-hoc Bonferroni-corrected *t-test*. The effect size was reported as the partial eta-square (*η^2^*). Bivariate correlations were conducted using Pearson product–moment correlations between demographic variables (age, years of education, BMI, and sex [coded as 1 = male, 2 = female]), aerobic fitness indices, and auditory oddball task performance measures.

Linear hierarchical regression analyses were then performed using attentional measures that were significantly correlated with aerobic fitness. Aerobic fitness measure was entered into step 2 in hierarchical regression analysis after the inclusion of demographic variables (i.e., age, education, and BMI) that were significantly correlated with attentional measures into step 1. Assumptions of linearity, equality of variance, independence, and normality were plotted, inspected, and verified using studentized residuals. A sensitivity analysis was conducted using G*Power 3.1.9.7, a widely used statistical tool for power analysis (Faul et al., 2007). Based on the current sample of 93 participants, with an alpha of 0.05 and power of 0.8, this analysis indicated that the present study was adequately powered to detect a small to medium effect size exceeding ƒ^2^ = 0.08 (Cohen, 2013) for the variance explained by aerobic fitness while accounting for three demographic variables (age, education, and BMI) in the hierarchical regression analysis. All data analyses were performed in SPSS v.21 (IBM Corp., Armonk, NY, USA), utilizing a familywise alpha level of *p* = 0.05 (Table 2).

**Table 2:** Behavioral and neuroelectric measures among exercise mode groups (mean ± SD).

	Open-skill (*n* = 31)	Closed-skill (*n* = 31)	Control (*n* = 31)
Response time (ms)	361.55 ± 46.84	386.35 ± 48.44	368.32 ± 39.98
Response accuracy (%)	98.71 ± 1.79	99.03 ± 1.52	98.61 ± 1.78
P3 amplitude (μV)	15.10 ± 1.90	14.86 ± 2.09	12.40 ± 2.39
P3 latency (ms)	321.29 ± 50.11	310.29 ± 41.55	313.29 ± 38.77

## Results

### Participant demographics and aerobic fitness

Subject demographic data and their levels of physical activity and fitness are provided in Table 1. A one-way ANOVA revealed non-significant differences in age, education, height, weight, and depression among the three groups (*p*s > 0.05). As expected, the participants in the two exercise groups had significantly more exercise experience (i.e., years of regular exercise, duration per session, and frequency per week), *F*s (2, 90) ≥ 138.85, *p* < 0.01, *η^2^* ≥ 0.76, and greater levels of physical activity (i.e., METs), *F* (2, 90) = 108.39, *p* < 0.01, *η^2^* > 0.71, and higher aerobic fitness, *F* (2, 90) = 96.60, *p* < 0.01, *η^2^* > 0.68, than those in the control group. Participants in the control group also had greater BMI, *F* (2, 90) = 9.47, *p* < 0.01, *η^2^* > 0.17, than those in the exercise groups. A *post hoc* comparison demonstrated non-significant differences between the open- and closed-skill exercise groups in these indices (*t*s ≥ 0.19, *p* > 0.05).

### Attentional function

**Figure 2:**
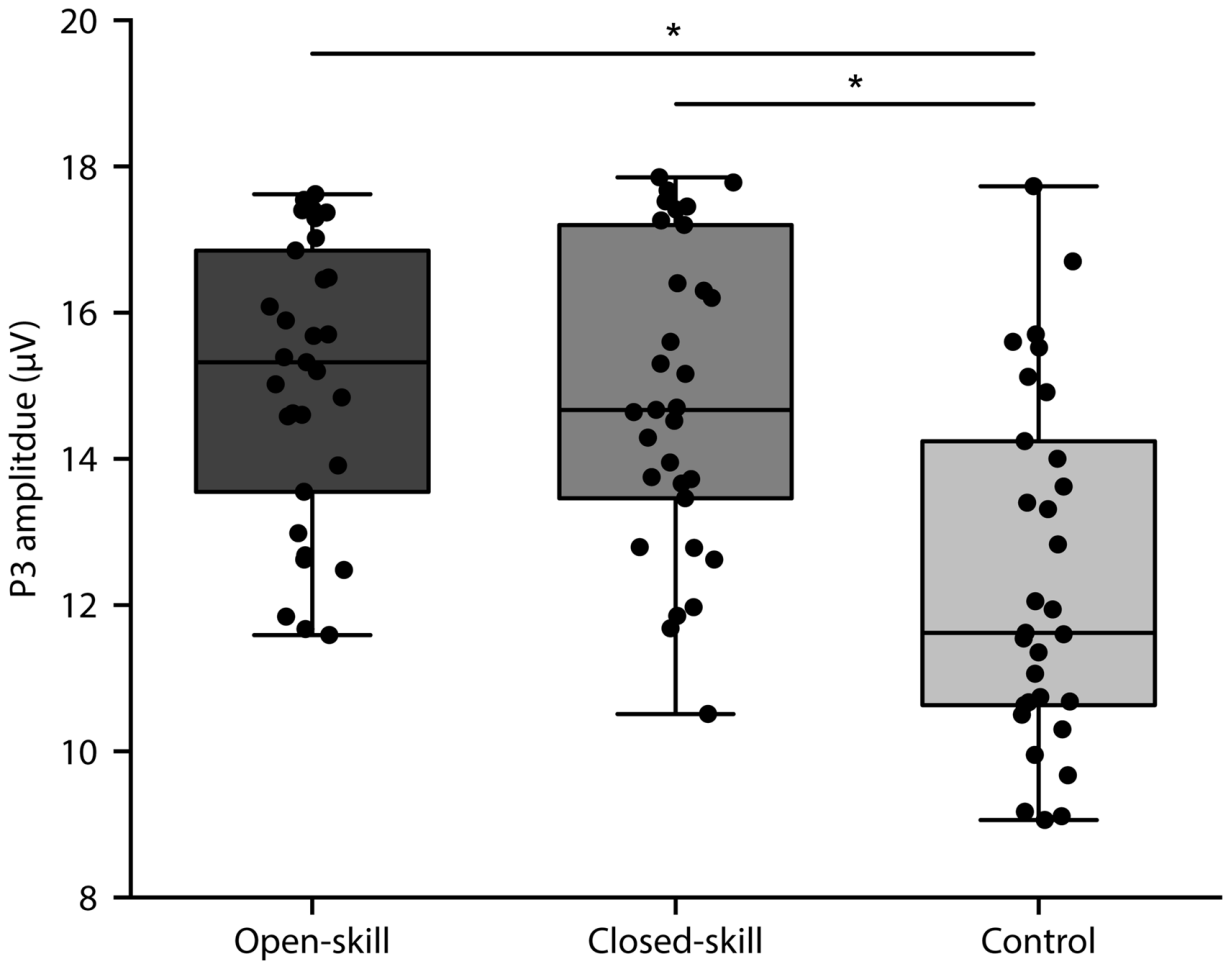
P3 amplitude across open-skill exercisers, closed-skill exercisers, and controls.

The behavioral and neuroelectric data of the three groups are presented in Table 2. A one-way ANOVA revealed non-significant differences in response time, response accuracy, and P3 latency among the three groups (*p*s > 0.05). Participants in the open-skilled and closed-skilled groups exhibited larger P3 amplitude, *F*s (2, 90) = 15.07, *p* < 0.01, *η^2^* ≥ 0.25, than those in the control group. A *post hoc* comparison demonstrated non-significant differences between the open- and closed-skill exercise groups in P3 amplitude (*t* = 0.46, *p* > 0.05). Figure 2 shows the mean P3 amplitude in the three groups.

### Bivariate correlations

Table 3 summarizes the results of the initial Pearson product–moment correlations among age, education, BMI, physical activity (IPAQ), aerobic fitness (YMCA), and behavioral and neuroelectric measures of attention (P3 component). The results show that aerobic fitness was associated with greater P3 amplitude (*r* = 0.48). Accordingly, P3 amplitude was used as a dependent variable in the regression models. P3 amplitude was significantly correlated with age, education, and BMI (*r*s ≥ 0.34). Because of these detected associations, age, education, and BMI were controlled in the regression analyses.

**Table 3:** Bivariate correlations between demographic factors, physical activity, aerobic fitness, and behavioral and neuroelectric measures of the oddball task.

	1	2	3	4	5	6	7	8	9	10	11	12	13	14
1. Sex														
2. Age	−0.06													
3. Education	−0.10	0.95**												
4. Height	−0.228*	0.06	0.06											
5. Weight	−0.231*	0.21*	0.16	0.61**										
6. MMSE	0.09	0.05	0.04	−0.03	−0.04									
7. BDI	−0.02	0.05	−0.01	0.02	0.05	−0.10								
8. BMI	−0.10	0.215*	0.15	−0.12	0.72**	−0.03	0.04							
9. YMCA	−0.20	−0.12	−0.03	0.17	−0.14	0.01	0.01	−0.33**						
10. IPAQ	−0.01	0.26*	0.31**	0.16	−0.07	−0.04	0.01	−0.23*	0.66**					
11. Response time	0.04	0.20	0.18	−0.06	−0.07	0.16	0.01	−0.04	0.09	0.19				
12. Response accuracy	−0.04	−0.13	−0.13	−0.05	−0.12	0.03	−0.08	−0.12	0.14	0.05	−0.02			
13. P3 amplitude	0.09	−0.47**	−0.40**	0.10	−0.20	−0.05	0.07	−0.34**	0.48**	0.121	−0.41**	0.07		
14. P3 latency	0.12	0.09	0.12	−0.24*	−0.10	0.14	0.16	0.09	−0.11	0.00	0.21*	0.03	−0.12	

### Hierarchical regression analyses

**Figure 3:**
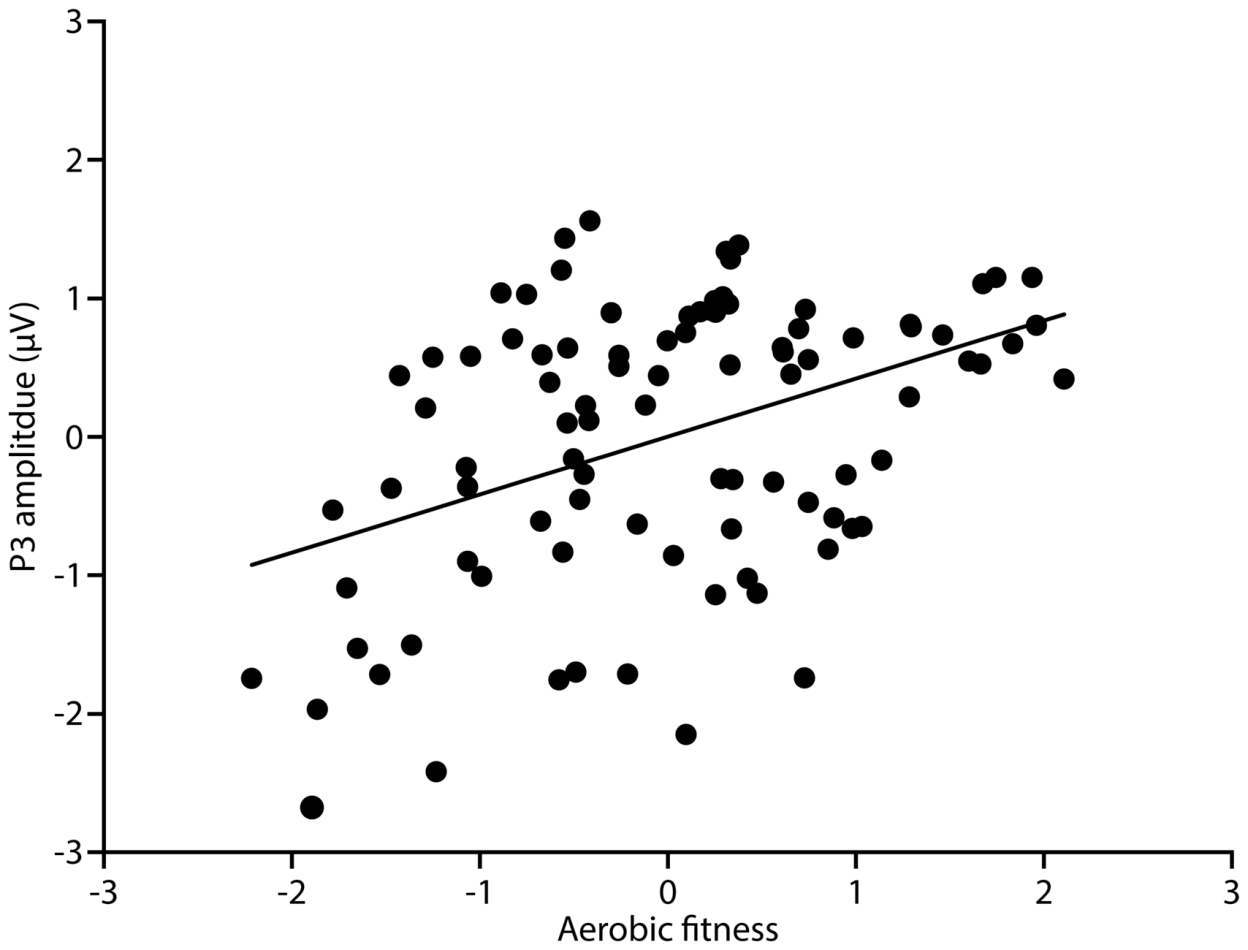
Scatterplots (*n* = 93) of the correlation between aerobic fitness and P3 amplitude after controlling for age, education, and BMI using studentized residuals.

Table 4 summarizes the hierarchical regression results for P3 amplitude. Figure 3 illustrates the association between aerobic fitness and P3 amplitude. The hierarchical regression analysis indicated that young adults with higher aerobic fitness exhibited greater P3 amplitude, *β* = 0.39, *t* = 4.32, *p* < 0.01, Cohens’ ƒ^2^ = 0.20, after controlling for age, education, and BMI (see Table 4 and Figure 3).

**Table 4:** P3 amplitude hierarchical regression values for aerobic fitness.

	*B*	SEB	*β*	*t*	*R* ^2^	Δ*R*^2^	Δ*F*
Step 1					0.26	0.29	11.85**
Age	−1.24	0.53	−0.70	−2.35*			
Education	0.59	0.58	0.30	1.02			
BMI	−0.30	0.12	−0.24	−2.54*			
Step 2					0.38	0.13	18.69**
Age	−0.73	0.50	−0.41	−1.47			
Education	0.04	0.54	0.02	0.08			
BMI	−0.16	0.11	−0.13	−1.44			
YMCA	0.07	0.02	0.39	4.32**			

*p < 0.05, **p < 0.01.

## Discussion

This study provides new insights into the association between motor skill experience and attentional functioning using behavioral and neuroelectric measures in healthy young adults. Specifically, we investigated whether cognitive benefits associated with sports differ based on the type of motor skills (open vs. closed) and whether these benefits are observable in non-sport-specific cognitive tasks. Our results indicate that motor skill experience, regardless of whether it involves open or closed skills, is associated with larger P3 amplitudes, suggesting enhanced attentional resource allocation. Additionally, hierarchical regression analysis confirmed a positive correlation between P3 amplitude and aerobic fitness, emphasizing the critical role of physical fitness in cognitive processing. These findings suggest that attentional enhancements related to sports can extend beyond the sports context, regardless of sport type, and highlight the importance of aerobic fitness in attentional functioning.

Contrary to our hypothesis, we found no significant differences in attentional measures (i.e., response time, response accuracy, P3 amplitude) between open-skill and closed-skill sports participants. This finding is consistent with studies that have shown similar effects on visuospatial attention and processing speed between open- and closed-skill exercises (Chueh et al., 2017; Giglia et al., 2011; Guo et al., 2016). However, other research has suggested that open-skill sports, with their dynamic and unpredictable nature, confer additional cognitive benefits, such as enhanced cognitive flexibility, inhibitory control, and decision-making (Ingold et al., 2020; Takahashi and Grove, 2023; Zhou et al., 2020). These differences are attributed to the increased cognitive demands of adapting to changing environments during open-skill sports compared to the repetitive and predictable nature of closed-skill exercises (Zhu et al., 2020). A systematic review further supports the idea that open-skill exercises are more effective in improving certain aspects of cognitive function compared to closed-skill exercises (Gu et al., 2019). The lack of significant differences in this study may be due to the specific attentional measures assessed, as both exercise types can enhance attention through different mechanisms—open-skill sports through adaptability to changing stimuli and closed-skill sports through focused, sustained attention. Accordingly, the specific cognitive domains being assessed might significantly influence the outcomes of studies comparing open-skilled and closed-skill exercises. Executive function tasks, which involve higher-order cognitive processes, are more likely to reveal differences between the two types of exercise due to the complex and adaptive nature of open-skilled activities.

The limited evidence for superior cognitive benefits of open-skill sports in young adults in the current study could also be attributed to the fact that cognitive functions typically peak during young adulthood, reducing the potential for further enhancements through sports training (Casey et al., 2000). Therefore, as such, the differences in cognitive benefits between open- and closed-skill sports may be more pronounced in populations with developing or declining cognitive abilities. Future studies should include long-term interventions across different age groups to better assess the potential differential effects of open- and closed-skill sports (Gu et al., 2019; Zhu et al., 2020).

Our study further demonstrated that both sports groups had larger P3 amplitudes compared to the control group, replicating findings from previous research linking sports participation to enhanced P3 amplitudes (Aly et al., 2019; Aly and Kojima, 2020b). The positive relationship between aerobic fitness and P3 amplitude observed in our regression analysis aligns with existing literature on the neurocognitive benefits of aerobic fitness (Aly et al., 2024; Luque-Casado et al., 2016; Wang et al., 2016; Wu et al., 2022). This is in line with a growing body of studies that indicate that increased P3 amplitude after moderate to vigorous aerobic exercise (Aly and Kojima, 2020a; Chang et al., 2017; Chang et al., 2015; Dimitrova et al., 2017; Kamijo et al., 2009). The P3 component is linked to attentional processing and working memory, with larger P3 amplitudes indicating better attentional resource allocation and more efficient cognitive processing (Reed et al., 2022; Steiner et al., 2016). Thus, higher levels of aerobic fitness may contribute to improved neural efficiency even in young adults, potentially due to physiological mechanisms such as increased cerebral blood flow (Stimpson et al., 2018), the proliferation of neurotrophic factors like BDNF (Cefis et al., 2023), and enhanced synaptic plasticity (Pavlović, 2016).

A key strength of our study is its contribution to the generalizability of the association between aerobic fitness, motor skill experience, and P3 amplitude across diverse populations. While existing evidence on the positive relationship between aerobic fitness, sports participation, and P3 has primarily focused on Western or Asian populations (Kao et al., 2020; Ludyga et al., 2020), our findings among Egyptian participants, who are Arabic native speakers, extend this association to a Middle Eastern context. This cross-cultural evidence supports the broader applicability of the cognitive benefits of aerobic fitness and sports participation, adding valuable insights into how these associations hold across different linguistic and cultural backgrounds.

Despite the valuable insights gained from this study, several limitations must be acknowledged. First, the cross-sectional design limits our ability to infer causality regarding the relationship between motor skill type and attentional processing. Longitudinal intervention studies are needed to establish causality and to understand the long-term effects of different types of sports engagements on cognitive functions. Second, although the auditory oddball task is a well-established measure of attentional processing, it may not fully capture the complexity of attentional engagement required in real-world scenarios. Future studies should incorporate assessments of different types of attention (e.g., divided attention, sustained attention) using neuroelectric measures to provide a more comprehensive evaluation of attentional functioning. Additionally, while presenting grand average waveforms for the P3 ERP component would enhance the visual representation of our data, this was not feasible with our EEG system, which provides only individual participant waveforms and lacks data extraction capabilities for external analysis. Future studies should consider EEG systems that support data extraction to enable group-averaged waveform presentations. Lastly, although we controlled for several confounding factors (age, education, and BMI), other variables that may influence cognitive performance, such as dietary habits (Shang et al., 2021), sleep quality (Wang et al., 2023), and genetic predispositions (Vo et al., 2022), were not accounted for in this study. Addressing these limitations in future research will help to further elucidate the complex relationship between physical exercise and cognitive health.

## Conclusion

This study advances our understanding of the relationship between motor skill experience, aerobic fitness, and attentional processing by examining these factors in a sample of young adults from an Egyptian population. Our findings demonstrate that both open- and closed-skill sports are associated with enhanced attentional engagement, as reflected by increased P3 amplitudes, suggesting that the attentional benefits derived from motor skill experience extend beyond the sports context itself. Additionally, the positive association between aerobic fitness and P3 amplitude underscores the role of physical fitness in supporting cognitive efficiency, highlighting aerobic fitness as a potential contributor to attentional enhancement. While no significant differences in attentional measures were observed between open-skill and closed-skill athletes, this study emphasizes the need for further research into how different types of sports may affect specific cognitive domains, particularly those requiring complex, adaptive attentional processing. Our findings, including participants from a Middle Eastern context, contribute to the generalizability of prior research largely conducted in Western and Asian populations, supporting a broader applicability of the cognitive benefits of motor skills and fitness. Future longitudinal studies should explore these relationships across diverse age groups and real-world cognitive tasks to deepen our understanding of how sports engagement and fitness shape cognitive health over time.

## Data Availability

The raw data supporting the conclusions of this article will be made available by the authors without undue reservation.
